# Taurus, an Important Diversification Center for the Genus *Aethionema* (Brassicaceae): Four New Species from the Central Taurus Mountains in Türkiye

**DOI:** 10.3390/plants15081180

**Published:** 2026-04-11

**Authors:** Kuddisi Ertuğrul, Tuna Uysal, Meryem Bozkurt, Emrah Şirin, Hakkı Demirelma, Burcu Yılmaz Çıtak

**Affiliations:** Department of Biology, Faculty of Science, Selçuk University, Konya 42130, Türkiye; tuysal@selcuk.edu.tr (T.U.); mbozkurt@selcuk.edu.tr (M.B.); emrahsirin@selcuk.edu.tr (E.Ş.); hdemirelma@selcuk.edu.tr (H.D.); burcuyilmaz@selcuk.edu.tr (B.Y.Ç.)

**Keywords:** Anatolia, Cruciferae, kayagülü, phylogeny, stonecress

## Abstract

This paper explores the role of the Taurus Mountains in shaping species differentiation and biogeographical regions, including distinct ecological zones at various altitudes. The genus *Aethionema* is a monophyletic group in tribe Aethionemeae, subfamily Aethionemoideae (Brassicaceae), a sister group to the rest of the family. *Aethionema* has significant taxonomic complexity, particularly in Türkiye, where the genus has the highest species diversity. In this study, four new species (*Aethionema kadriyeae*, *A. uysalii*, *A. beysehirense*, and *A. ermenekense*) are described based on morphological, palynological and phylogenetic analyses. The diagnoses, detailed descriptions, distribution maps, and illustrations of the new species are provided. Pollen and seed morphology, including detailed measurements of size, ornamentation, and shape, is given. Phylogenetic analyses using DNA sequences from nuclear region (ITS) and chloroplast region (*rpl32-trnL^UAG^*) were conducted to determine the evolutionary relationships within the genus. Overall, this research provides new insights into the biodiversity and evolutionary history of *Aethionema* in Türkiye, highlighting the significance of the Taurus Mountains in supporting rich ecological diversity.

## 1. Introduction

Türkiye is notable for its remarkable species diversity, hosting three biodiversity hotspots within the Palearctic region: the Mediterranean, the Caucasus, and the Irano-Anatolian regions. Positioned at the crossroads of Asia, Europe, and Africa, Anatolia benefits from a unique geographic location that has contributed to its rich biological diversity. Over the course of glacial and interglacial periods, various floral and faunal elements (e.g., Siberian, African, Mediterranean, Boreal, and Eremial) have migrated into Anatolia, enriching its ecological landscape. These periods also facilitated the formation of numerous refugia, further enhancing the region’s biodiversity. Among Anatolia’s key ecological features, the Taurus Mountains play a particularly significant role in supporting and preserving its biological richness [1].

The species diversity observed along the Mediterranean phytogeographic region and the Anatolian Diagonal can be associated with past tectonic events. The belt was proposed as one of the most significant topographic barriers between the eastern and western parts of Anatolia through delimiting distributional ranges of many species. Similar to the formation of the Alborz, Zagros and Kopeh-Dag Mountain ranges in Iran, the formation of the Taurus and Pontic Mountain ranges in Türkiye is part of the Alpine orogeny caused by the collision of the African and Arabian plates with the Eurasian plate [2,3,4]. In particular, the collision of the Arabian and Eurasian plates during the Miocene accelerated the uplift of the Anatolian plateaus and allowed the formation of many different habitat types between the Mediterranean and the formed plateaus [5]. The formation and uplift of these areas caused the aridification of the Irano-Turanian region, turning the surrounding regions into a melting pot of climatic characteristics [5,6]. This has made the Irano-Turanian and Mediterranean regions in Türkiye a home where species boundaries intersect, coexist, and hybridize. In addition, orobiomes and different altitudinal boundaries in different parts of the Taurus Mountains have allowed differentiation of different taxa [7].

Mountain ranges exceeding 1000 m in elevation within the same climatic zone often lead to vertical ecological zonation. This stratification typically supports three distinct orobiomes. For example, in the Taurus Mountains, the lower zone, ranging from the coast to approximately 1000–1200 m, serves as the primary habitat for typical Mediterranean vegetation, including Calabrian pine (*Pinus brutia* Ten.) and maquis communities. The middle zone, situated between 1200 and 2000 m, is characterized by forests dominated by cedar (*Cedrus libani* A.Rich.), Taurus fir (*Abies cilicica* (Antoine & Kotschy) Carrière) and Anatolian black pine (*Pinus nigra* J.F.Arnold). Pure stands of Calabrian pine forest grow only under Mediterranean climate conditions characterized by mild rainy winters and hot and dry summers, while cedar, fir and Anatolian black pine grow under cold and snowy winters and cool and slightly rainy summers. Therefore, the boundary extending between the two different forest belts is considered the main orobiome boundary of the Taurus Mountains. Above this, the upper zone corresponds to a subalpine region, where alpine and steppe plant species are found [7,8]. The mountain ranges of Anatolia, particularly the Taurus Mountains, play a pivotal role in speciation and in delineating biogeographical subregions and provinces. These ranges, characterized by numerous valleys, especially in the southern regions, significantly shape the area’s biodiversity. The Cilician and western Taurus largely define the East Mediterranean province, while the southeastern Taurus serves as a boundary between the Irano-Anatolian and Mesopotamian provinces. Additionally, the Pontic Taurus facilitates the exchange of Irano-Anatolian elements with Euxinic elements, whereas the eastern Taurus creates pronounced distinctions between central and eastern Anatolia [9]. All four new *Aethionema* species grow in the middle section of the Taurus Mountains along the “middle zone” line of the Mediterranean vegetation.

As the sister group to the rest of Brassicaceae, *Aethionema* W.T. Aiton is the sole genus in tribe Aethionemeae and subfamily Aethionemoideae [10]. The complexity of *Aethionema* arises from the limited number of macro-morphological traits, such as those related to fruits and leaves, that can be used to distinguish species. *Aethionema* is a taxonomically difficult genus representing 68 species worldwide [11], and its center of highest diversity is Türkiye, with fewer species in neighboring Iran, Caucasus, and Greece. The distribution of individual species extends eastward to Kazakhstan and westward to Spain and Morocco [12,13,14]. The genus *Aethionema* was represented by 30 species in the first volume of the Flora of Turkey [15]. Ertuğrul [16] recognized 40 species of *Aethionema* in Türkiye, but subsequent discoveries increased it to 57 [17,18,19,20,21,22,23]. Following recent taxonomic revisions to the genus, the number of species in Türkiye was reduced to 47 [24].

This study aims to investigate the influence of the Taurus Mountains on species differentiation and biogeographical structuring by describing four new species of *Aethionema* from Türkiye. Utilizing an integrative approach that combines morphological, palynological, and phylogenetic analyses, this research seeks to resolve the taxonomic complexity within the genus, clarify evolutionary relationships, and contribute new insights into the biodiversity and evolutionary history of *Aethionema* in one of its primary centers of diversity. As a result of the completed systematic and phylogenetic studies, numerous samples belonging to various species were collected during recent extensive fieldwork. Following comprehensive herbarium studies, including all type collections, it was concluded that these samples represent four new species, hereafter recognized as *Aethionema. kadriyeae* Ertuğrul, *A. uysalii* Ertuğrul & Demir., *A. beysehirense* Ertuğrul & Şirin, and *A. ermenekense* Ertuğrul & Uysal.

## 2. Results

### 2.1. Palynomorphological Characteristics

All studied species possess radially symmetrical, isopolar pollen grains, with tricolpate as the predominant aperture type; however, *Aethionema armenum* Boiss., *A. spicatum* Post, and *A. yildirimlii* Kılıç additionally exhibit tetracolpate pollen, whereas *A. kadriyeae* displays syncolpate grains. In all taxa, the pollen outline is triangular or orbicular in polar view and elliptic in equatorial view, and the colpus is long and sunken and possesses distinct, regular margins with an ovate end (Figure 1). Pollen shape and size vary considerably among species: *A. kadriyeae* exhibits prolate-spheroidal pollen (P: 20.01 ± 0.90 μm, E: 12.42 ± 0.42 μm) with a microreticulate exine and narrow muri, whereas *A. turcicum* H.Duman & Aytaç has prolate pollen (P: 20.04 ± 2.31 μm, E: 13.21 ± 1.79 μm) and a reticulate exine with wider muri. Among *A. uysalii*, *A. coridifolium* DC., and *A. spicatum*, pollen shape is consistently prolate-spheroidal, yet aperture morphology differs: *A. uysalii* and *A. coridifolium* are tricolpate, while *A. spicatum* exhibits both tricolpate and syncolpate grains, with pollen dimensions of P: 17.64 ± 1.13 μm, E: 15.24 ± 1.85 μm; P: 16.50 ± 1.16 μm, E: 15.75 ± 0.82 μm; and P: 20.44 ± 1.00 μm, E: 20.22 ± 0.46 μm, respectively, and all three possess reticulate pollen but differ in muri size (Figure 1 and Table 1). *Aethionema beysehirense* exhibits subprolate pollen (P: 21.14 ± 2.41 μm, E: 16.91 ± 3.45 μm) with a reticulate exine, whereas *A. armenum* (P: 18.12 ± 0.78 μm, E: 16.23 ± 0.50 μm) and *A. schistosum* (P: 16.41 ± 1.04 μm, E: 13.66 ± 1.03 μm) are prolate-spheroidal and microreticulate, with all three varying in muri size (Table 1). Finally, *A. ermenekense*, *A. spicatum*, and *A. yildirimlii* share prolate-spheroidal pollen, with dimensions of P: 17.64 ± 1.13 μm, E: 15.24 ± 1.85 μm; P: 20.44 ± 1.00 μm, E: 20.22 ± 0.46 μm; and P: 15.98 ± 0.99 μm, E: 15.56 ± 0.87 μm, respectively, and exine ornamentation varies from microreticulate in *A. ermenekense* to reticulate in *A. spicatum* and *A. yildirimlii*, with corresponding differences in muri size (Figure 1 and Table 1).

### 2.2. Seed Morphology

The investigated *Aethionema* taxa have ovate to broadly ovate seeds with obtuse base and rounded apex and vary in color from light-brown to brown, though *A. ermenekense* differs from the rest by having brown-grey seeds. The common seed sculpture is reticulate-verrucate. The seed size of *A. kadriyeae* is 2.17 × 1.26 mm and of *A. turcicum* is 1.65 × 0.86 mm (Figure 2, Table 2), and both have ovate seeds with a striate pattern. Seed sizes are 2.11 × 1.42 mm in *A. uysalii*, 2.29 × 1.25 mm in *A. spicatum*, 2.87 × 1.73 mm in *A. coridifolium*, 2.33 × 1.25 mm in *A. beysehirense*, 2.11 × 1.00 mm in *A. armenum*, 2.02 × 1.18 mm in *A. schistosum*, 2.79 × 1.49 mm in *A. ermenekense*, and 2.68 × 1.47 mm in *A. yildirimlii* (Table 2). The largest seeds are found in *A. ermenekense*, and the smallest are found in *A. turcicum*. The cells of seed-surface testa are orbicular with irregular epidermal cells (Figure 2 and Figure 3). *A. kadriyeae*, *A. coridifolium*, *A. schistosum*, and *A. yildirimlii* have broadly ovate seeds that vary in epidermal cell size. Both *A. kadriyeae* and *A. turcicum* have regular cells, though the anticlinal walls are clearer in the former. The verrucae of epidermal cells are convex in all the species studied except *A. yildirimlii* (Figure 2 and Table 2).

### 2.3. Phylogenetic Studies

The ITS and *rpl32-trnL* dataset includes sequences of 14 *Aethionema* taxa. The length of the aligned datasets is 617 (ITS) and 636 (*rpl32-trnL*^UAG^) base pairs. PAUP and MrBayes programs were used to construct phylogenetic trees, and tree topologies in NJ, MP, ML and BI were similar. Due to such similarities, the following discussion will focus only on NJ (Figure 4). Based on the ITS sequence data, the Neighbor-Joining (NJ) tree resolved the *Aethionema* taxa into two major clades. The first main clade, comprising *A. turcicum*, *A. kadriyeae*, *A. dumanii*, and *A. aytachii*, is strongly supported (PP = 1.00; BS = 94% for MP/95% for ML). The remaining *Aethionema* taxa are grouped within the second main clade. Similarly, the NJ tree constructed from the *rpl32-trnL* sequence data also divided the *Aethionema* taxa into two main clades. The first main clade includes *A. kadriyeae*, *A. dumanii*, and *A. aytachii*. The second main clade is further subdivided into two subclades: the first subclade consists solely of *A. turcicum* (PP = 0.82; BS = 68 for MP/67 for ML), while the second subclade contains the remaining *Aethionema* taxa and is strongly supported (PP = 0.93; BS = 93 for MP/87 for ML) (Figure 4). In the phylogenetic trees, certain taxa (such as *A. ermenekense*, *A. yildirimli*, and *A. spicatum* for ITS and *A. yildirimli* and *A. coridifolium* for *rpl32-trnL*) remain unresolved, forming partial polytomies. These patterns suggest the presence of complex evolutionary relationships that cannot be fully captured by tree-based approaches. These complex evolutionary relationships observed in the trees were resolved using network analyses.

### 2.4. Taxonomy

#### 2.4.1. *Aethionema kadriyeae* Ertuğrul, sp. Nov. (Figure 5 and Figure 6)

*Type*: Türkiye. Karaman: Kayaönü village, south slopes of Karadere, marly steppe, 1620 m, 19 May 2021, *K. Ertuğrul* 6339 and *B. Yılmaz Çıtak* (holo KNYA; isotypes GAZI, ANK).

**Figure 5:**
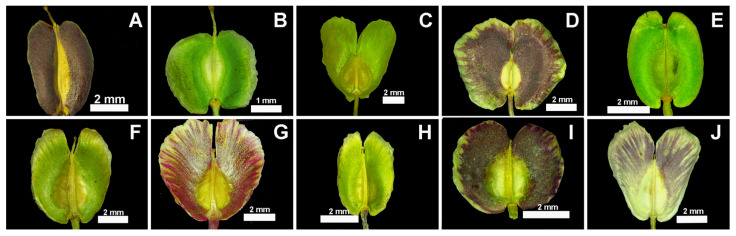
The fruit light microscope microphotographs of examined *Aethionema* species. (**A**) *A. kadriyeae*, (**B**) *A. turcicum*, (**C**) *A. uysalii*, (**D**) *A. spicatum*, (**E**) *A. coridifolium*, (**F**) *A. beysehirense*, (**G**) *A. schistosum*, (**H**) *A. armenum*, (**I**) *A. ermenekense*, (**J**) *A. yildirimlii*.

**Figure 6:**
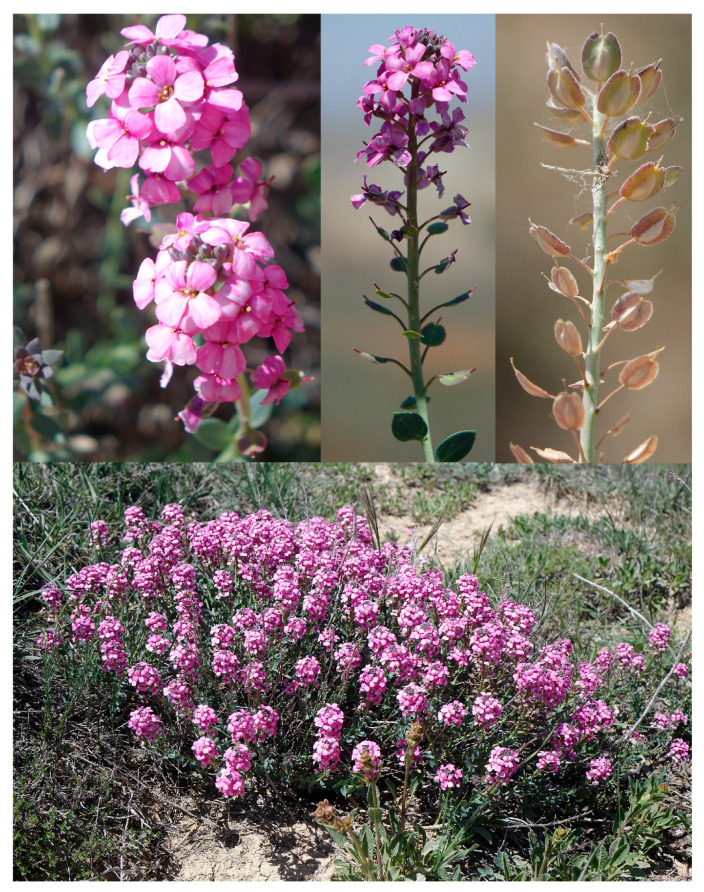
Habit, flowers and infructescence of *Aethionema kadriyeae*.

*Diagnosis: A. kadriyeae* is similar to *A. turcicum*, but it differs by its elliptic ovary (vs. oblong), edentate inner filaments (vs. dentate), 6–8 × 5.5–7 mm fruits (vs. 8.5–11 × 8–9), cordate base (vs. sagittate) at base, 1.5–2.5 mm length wing (vs. 2.5–3 mm) and open (vs. closed) sinus (Table 3).

Perennial, woody at base, many stemmed; stems 7–21 cm long, erect–ascending, sometimes branched towards the apex, glabrous, glaucous. Upper stem leaves sub-fleshy, glaucous, subsessile, alternate, elliptic, attenuate at base, obtuse and mucronulate at apex, 5.5–9 × 3–5.5 mm, lower stem leaves sub-fleshy, glaucous, subsessile, alternate rarely opposite, ovate, attenuate–rounded base, obtuse at, 4.5–5.5 × 2–3 mm, glomerule leaves more prominent during fruiting period, sub-fleshy, glaucous, subsessile, rosulate–alternate, oblong–elliptic, attenuate, acute at apex, 2–5 × 0.7–1.5 mm. Racemes 1–3 cm long, cylindrical, elongated in fruit. Pedicels 4–5 mm long in flowers, 5–5.5 mm in fruit, erect. Sepals oblong, violet margined with green band, 2.5–3.5 × 1.5–1.6 mm. Petals obovate, truncate, 6.5–7.5 × 2.5–4 mm, purple, claw and blade not distinct. Filaments free; inners not toothed, dilated at base, 3–3.5 mm long; outers 1.8–2.1 mm long; anthers yellow, apiculate, 0.5–0.8 mm long; stigma capitate; ovary elliptic. Infructescence lax, 3–9 cm long; fruit weakly cymbiform, dehiscent, oblong–obovate, 6–8 × 5.5–7 mm, bilocular, 2-seeded per locule, cordate at base, cell 3.5–5.5 × 2–2.5 (–3.5) mm, septum 4–5.5 × 1–2 mm, undulate at margin, wing 1.5–2.5 mm long, sinus open and 0.5–0.8 mm deep, style 1–1.7 mm long. Seed light-brown or brown, 2.148–2.205 × 1.24–1.28 mm, broadly ovate, verrucate.

*Etymology*: This new species is named in honor of the first author’s wife Kadriye Ertuğrul for her contribution to most fieldwork. The vernacular name of *A. kadriyeae* was proposed as “Sultan kayagülü” according to guidelines published by Menemen et al. [25].

*Distribution*, *habitat and ecology*: *A. kadriyeae* grows on marly steppes around Kayaönü in the Ayrancı district of Karaman Province at altitudes of 1260–1680 m (Figure 7).

*Additional examined specimens*: *A. kadriyeae*: Türkiye. Karaman: Ayrancı, Kayaönü, south slopes of Karadere, marly steppe, 1620 m, 4 July 2021, *K. Ertuğrul* 6616 (KNYA); 20 May 2022, *K. Ertuğrul* 6815 (KNYA). Türkiye. Karaman: Between Küçükkoraş-Kayaönü villages, white marly slopes, 1600 m, 20 June 2012, *K. Ertuğrul* 4587 *& T. Uysal* (KNYA). Ayrancı, Kayaönü village, *Quercus* openings, limestone soils, 19 June 2013, *T. Uysal* 3055 *& al*. (KNYA).

*A. turcicum*: Ankara Ayaş road, Aysantı pass, marly slopes, 1180 m, 25 May 2021, *K. Ertuğrul* 6379 *& E. Şirin* (KNYA).

The infructescence and fruit shape of *A. kadriyeae* resemble those of *A. turcicum* but differ by its basally attenuate (vs. rounded) upper leaves, erect (vs. horizontal and recurved) fruiting pedicels, edentate inner filaments 3–3.5 mm long (vs. dentate and 2–3 mm long), outer filaments 1.8–2.1 mm long (vs. 1.3–1.8 mm), elliptic (vs. oblong) ovary, fruits 6–8 × 5.5–7 mm (vs. 8.5–11 × 8–9 mm), with cordate (vs. sagittate) fruit base, locule 3.5–5.5 mm (vs. 5.5–6.5 mm) long, septum 4–5.5 mm (vs. 5–7 mm) long, wing 1.5–2.5 mm (vs. 2.5–3 mm) long, and open (vs. closed) sinus.

#### 2.4.2. *Aethionema uysalii* Ertuğrul & Demir., sp. Nov. (Figure 5 and Figure 8)

*Type*: Türkiye. Antalya: Akseki, between Salamut-Morca plateaus, stony slopes, 1987 m, 8 July 2020, *K. Ertuğrul* 6124 *& E. Şirin* (holo KNYA; isotypes GAZI, ANK).

**Figure 8:**
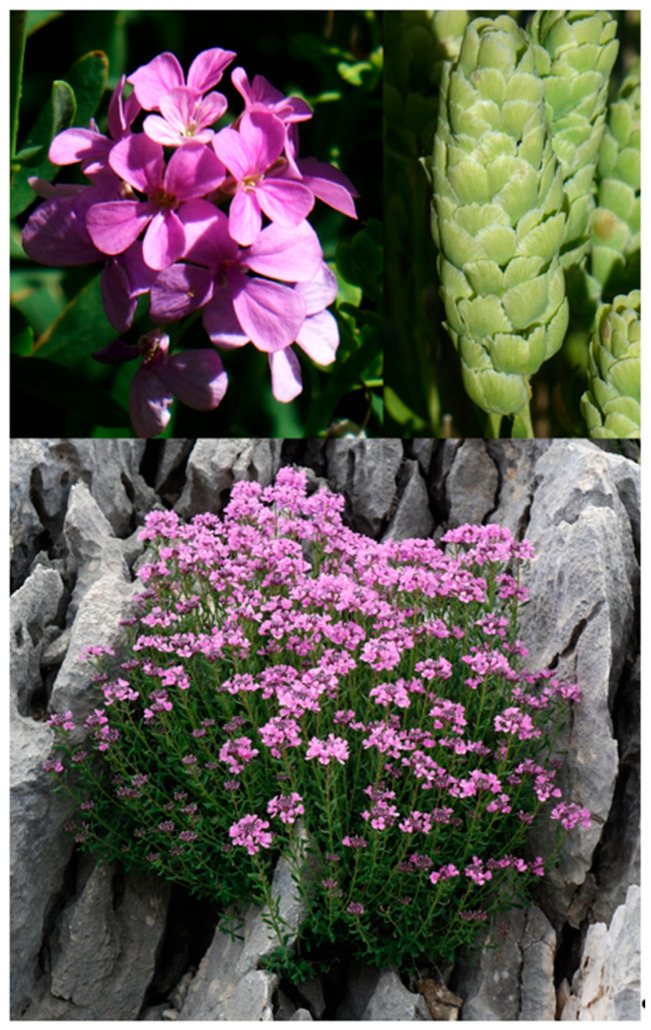
Habit, flowers and infructescence of *Aethionema uysalii*.

*Diagnosis*: *A. uysalii* is similar to *A. spicatum* and *A. coridifolium* but differs from *A. spicatum* by its oblanceolate (vs. obovate) stem leaves, 7.5–8 × 2.7–5 mm (vs. 4–6 × 1.5–2.1 (–3) mm) petals, and elliptic (vs. obovate) ovary. It differs from *A. coridifolium* by its capitate (vs. cylindrical) raceme, compact (vs. lax) infructescence, 8–11 mm (vs. 5.5–6.5 mm) fruit width, and 0.5 mm (vs. 0.2–0.3 mm) long styles (Table 3).

Perennial, with woody caudex, multi-stemmed at the base; sterile shoots, 10.5–36 cm long, erect, unbranched, glabrous, and glaucous. Stem leaves not fleshy, glaucous, subsessile, alternate, oblanceolate, attenuate at base, obtuse and sometimes mucronulate at apex, 12–25 × 2–4.5 mm; leaves of sterile stems glaucous, petiolate, opposite rarely alternate, oblanceolate, attenuate at base, obtuse at apex (2.5-) 4–10 × 1.5–3 mm. Racemes capitate, 0.7–2 cm long, elongated in fruit. Pedicels 3–4 mm long in flowers, 4.5–5 mm in fruit, adpressed. Sepals oblong, white margined with violet band, 3–3.5 × 0.8–1.2 mm. Petals spatulate–obovate, 7.5–8 × 2.7–5 mm, pink, claw and blade not distinct. Filaments free; inners not toothed, dilated at base, 1.8–2.8 mm long; outers 1–1.5 mm long; anthers yellow, truncate, 0.5–0.9 mm long; stigma capitate, papillose; ovary elliptic. Infructescence compact, imbricated, 1.5–5 cm long; fruits cymbiform, dehiscent, obovate, 7.5–9.5 × 8–11 mm, bilocular, 1-seeded per locule, cordate at base, cell 3–4 × 2.5–3.5 mm, septum 3.5–4 × 1–1.3 mm, undulate at margin, wing dilated towards apex and 4.5–5.5 mm long, sinus open and (3.5-) 4–5 mm deep, style 0.5 mm long. Seed light-brown, ovate, 2.77–2.82 × 1.48–1.51 mm, verrucate.

*Etymology*: This new species is named in honor of Süleyman Uysal, an amateur photographer interested in wild flowering plants in Antalya. The vernacular name of *A. uysalii* was proposed as “Akseki kayagülü” according to guidelines published by Menemen et al. [25].

*Distribution*, *habitat and ecology*: *A. uysalii* grows on rock crevices and stony slopes around Salamut-Morca plateaus in the Akseki district of Antalya province at altitudes of 1450–1987 m (Figure 7).

*Additional examined specimens*: *A. uysalii*: Türkiye. Antalya: Akseki, environs of Morca plateau, rocky areas, 1965 m, 8 July 2020, *K. Ertuğrul* 6140 *& E. Şirin* (KNYA); 6 August 2020, *H. Demirelma* 3444 *& E. Şirin* (KNYA). Türkiye. Antalya: Akseki, Fersin plateau, Evlek pass, rock crevices, 1710 m, 6 July 2020, *H. Demirelma* 3441 *& E. Şirin* (KNYA). Evlek pass, rocky areas, 1718 m, 8 July 2020, *K. Ertuğrul* 6123 *& E. Şirin* (KNYA). Akseki, Murtiçi, transmitter road of Gülen Mountain, rocky areas, 1450 m, 27 May 2022, *K. Ertuğrul* 6866 (KNYA). Akseki, Güzelsu, Gidefi Mountain, Gülek location, cliffs, 1733 m, 27 May 2022, *K. Ertuğrul* 6869 (KNYA).

*A. coridifolium*: Türkiye. Kahramanmaraş: Göksun, above Değirmendere, around Şarlak, stony slopes, 5 June 2022, *K. Ertuğrul* 6940 *& E. Şirin* (KNYA), Mersin, Ayvagediği, above Çandır Castle, around pond, clearings of *Pinus* forest, 1110 m, 27 June 2019, *K. Ertuğrul* 2940 (KNYA).

*A. spicatum*: Türkiye. Adana, Pozantı, Hamidiye to Çamlıbel, clearings of *Pinus* forest, 1290 m, 7 June 2020, *K. Ertuğrul* 6017 *& H. Demirelma* (KNYA), Mersin, Kaynakkeşli village, around Kızılasma bridge, clearings of *Pinus* forest, 760 m, 17 May 2019, *K. Ertuğrul* 5693 *& H. Demirelma* (KNYA).

*A. uysalii* resembles *A. spicatum* in having capitate racemes and compact infructescence and *A. coridifolium* in the linear–oblanceolate stem leaves. The novelty differs from *A. spicatum* by its non-fleshy, oblanceolate stem leaves and up to 25 mm long (vs. fleshy, obovate, up to 14 mm long) that are petiolate, oblanceolate on sterile stems (vs. subsessile, obovate), adpressed (vs. ascending) fruiting pedicels, petals 7.5–8 × 2.7–5 mm (vs. 4–6 × 1.5–2.1 (–3) mm), and elliptic (vs. obovate) ovary. It differs from *A. coridifolium* by having outer filaments 1–1.5 mm long (vs. 1.5–2.1 mm), elliptic (vs. circular) ovary, compact (vs. lax) infructescence, weakly cymbiform (vs. strongly cymbiform) fruits 8–11 mm (vs. 5.5–6.5 mm) wide, septum width 1–1.3 mm (vs. 1.4–2.5 mm), wing 4.5–5.5 mm long (vs. 2–3.5 mm), sinus 4–5 mm deep (vs. 1–2.5 mm) and style 0.5 mm long (vs. 0.2–0.3 mm).

#### 2.4.3. *Aethionema beysehirense* Ertuğrul & Şirin, sp. Nov. (Figure 5 and Figure 9)

*Type*: Türkiye. Konya: Konya-Beyşehir road, Gündoğdu-Gönen junction area, calcareous slopes, 1163 m, 12 July 2022, *K. Ertuğrul* 7063 (holo KNYA, isotypes GAZI, ANK).

*Diagnosis: A. beysehirense* is similar to *A. schistosum* and *A. armenum*, but it differs from *A. schistosum* by having obovate (vs. orbicular) fruits 5–7.5 mm (vs. 9–12 mm) wide and styles more than 0.5 mm (vs. less than 0.5 mm). It differs from *A. armenum* by its spatulate (vs. oblanceolate-obovate) petals and fruits more than 5 mm (vs. less than 5 mm) wide, and wings with closed sinus and more than 2 mm long (vs. open sinus and less than 2 mm long) (Table 4).

Perennial, with woody branches at the base; stems 5–22 cm long, erect–ascending, glabrous. Leaves heteromorphic: upper ones glaucous subsessile, alternate, linear, rarely involute and falcate, attenuate at base, usually acute at apex, 5.5–13 (–16) × 1–1.5 mm; glomerule leaves more prominent during fruiting period, glaucous, subsessile, rosulate-opposite rarely alternate, linear–oblong rarely oblanceolate, attenuate at base, acute, rarely obtuse at apex, 2–5 (–7) × 0.5–1 mm. Racemes cylindrical, 0.5–2.5 cm long, elongated in fruit. Pedicels 3–4.5 mm long in flowers, 4–6 mm in fruit, adpressed and recurved. Sepals oblong or rarely obovate, white margined with green band, rarely violet margined, 2.5–3.5 × 1–1.5 mm. Petals spatulate, 5–7 × 2–4 mm, violet, claw and blade almost distinct. Filaments free; inners not toothed, dilated at base, 1.7–2.7 mm long; outhers 1–1.6 mm long; anthers yellow, apiculate, 0.5–0.8 mm long; stigma capitate; ovary obovate. Infructescence lax, 1–7.5 cm long; fruit weakly cymbiform, dehiscent, obovate, 6.5–7.5 × 5–7.5 mm, bilocular, 1-seeded per locule, cordate at base, cell 3–4.5 × 2–3 mm, septum 3.5–5 × 1–1.6 mm, irregular dentate at margin, wing 2–4 mm long, sinus closed and 0.8–1 mm deep, style 0.5–1 mm long. Seed light-brown, ovate, 2.02–2.03 × 0.97–0.99 mm, verrucate.

*Etymology*: The specific epithet was derived from the name of Beyşehir district from where the first specimens of the new species were collected. The vernacular name of *A. beysehirense* was proposed as “Beyşehir kayagülü” according to guidelines published by Menemen et al. [25].

*Distribution*, *habitat and ecology*: *A. beysehirense* grows on gypsum, marly and limestone slopes on the roadside of the Konya to Beyşehir, Gündoğdu-Gönen junction area, on the roadside of Hüyük to Konya, Konya, Kızılören Mountain foothills, around Halkapınar village of Ereğli in Konya, west of Büyükkarapınar village of Başyayla in Karaman, and south of Ulukışla in Niğde, at altitudes of 1163–1683 m (Figure 7).

*Additional examined specimens*: *A. beysehirense*: Türkiye. Konya: Beyşehir road, Gündoğdu junction area, calcareous slopes, 1163 m, 3 June 2022, *K. Ertuğrul* 6905 (KNYA); Konya-Beyşehir road, Gündoğdu-Gönen junction, slopes, 1163 m, 22 May 2022, *K. Ertuğrul* 6853 (KNYA). Türkiye. Konya: Beyşehir, Hüyük-Konya road, after Selki, limestone slopes, 1232 m, 29 May 2022, *K. Ertuğrul* 6896 (KNYA); 12 July 2022, K. Ertuğrul 7062 (KNYA); Konya, Kızılören, above Kent Forest, Kızılören Mountain foothills, openings of *Juniperus*, 25 June 2021, *K. Ertuğrul* 6608 (KNYA); Ereğli, Halkapınar, Kayasaray village, Düğünlük stream, Kocaoluk area, limestone slopes 1650 m, 15 May 2022, *H. Demirelma* 3453 *& E. Şirin* (KNYA). Entrance of Düğünlük stream, *Quercus* openings, 1630 m, 25 May 2019 *K. Ertuğrul* 5747 *& E. Şirin* (KNYA); 12 July 2019, *K. Ertuğrul* 5910 *& al*. (KNYA); Ereğli, 5–10 km southwest of Büyükdede village, clearings of *Juniperus* forest, 1810 m, 20 June 2017, *K. Ertuğrul* 5386 *& H. Dural* (KNYA). Ereğli, Halkapınar, Kayasaray village, Karaman: Ermenek, Tekeçatı to Balkusan, limestone slopes, *Juniperus* and *Pinus* openings, 1620 m, 20 May 2022, *K. Ertuğrul* 6819 (KNYA); Ermenek, west of Büyükkarapınar village, limestone slopes, 1594 m, 21 May 2022, K. Ertuğrul 6824 (KNYA); 7 July 2022, *K. Ertuğrul* 7053 (KNYA), 1683 m, 2 July 2022, *H. Demirelma* 3471 *& E. Şirin* (KNYA); 1610 m, 15 July 2019, *K. Ertuğrul* 5918 (KNYA);1685 m, 2 July 2022, H. *Demirelma* 3469*A & E. Şirin* (KNYA); Niğde: South of Ulukışla, around Evrenhasan Tepe, eroded clayey slopes, 1600 m, 8 May 2022, *K. Ertuğrul* 6801 (KNYA); 1560 m, 15 June 2017, *K. Ertuğrul* 5337 *& H. Dural* (KNYA); *Cedrus* plantation area, 1550 m, 12 July 2019, *K. Ertuğrul* 5883 *& al*. (KNYA); gypsum and limestone slopes, 1670 m, 12 July 2019, *K. Ertuğrul* 5888, 5888A *& al*. (KNYA); 1700 m, 12 July 2019, *K. Ertuğrul* 5894 *& al*. (KNYA).

*A. schistosum*: Türkiye: Konya, Seydişehir, west of Madenli willage, slopes, 1710 m, 4 July 2019, *K. Ertuğrul* 5863 (KNYA), Adana: Tufanbeyli to Tomarza, between Ayvat village and Tomarza, stony slopes, 2003 m, 14 June 2022, *K. Ertuğrul* 7036 *& E. Şirin* (KNYA), Adana: Pozantı to Çamardı, between Fındıklı and Kamışlı villages, *Pinus* openings, 1200 m., 8 May 2022, *K. Ertuğrul* 6797 (KNYA).

*A. armenum*: Kayseri, Alidağı, west foothills, stony slopes, 1378 m, 12 June 2021, *K. Ertuğrul* 6513 *& E. Şirin* (KNYA); Kayseri, Pınarbaşı, between Aşağıbeyçayırı and Yukarıbeyçayırı villages, serpentine slopes, 12 June 2021, *K. Ertuğrul* 6524 *& E. Şirin* (KNYA).

*A. beysehirense* resembles *A. schistosum* in having linear stem leaves and spatulate petals and *A. armenum* in the shape of stem leaves and lax infructescence. It differs from *A. schistosum* by having obovate (vs. orbicular) fruits 5–7.5 mm (vs. 9–12 mm) long, with fruit wing less than 4 mm (vs. more than 4 mm) long, and style more than 0.5 mm (vs. less than 0.5 mm) long. It differs from *A. armenum* by having spatulate (vs. oblanceolate–obovate) petals differentiated (vs. undifferentiated) into claw and blade, and fruits more than 5 mm (vs. less than 5 mm) wide with wing more than 2 mm (vs. less than 2 mm) long and closed (vs. open) sinus.

**Figure 9:**
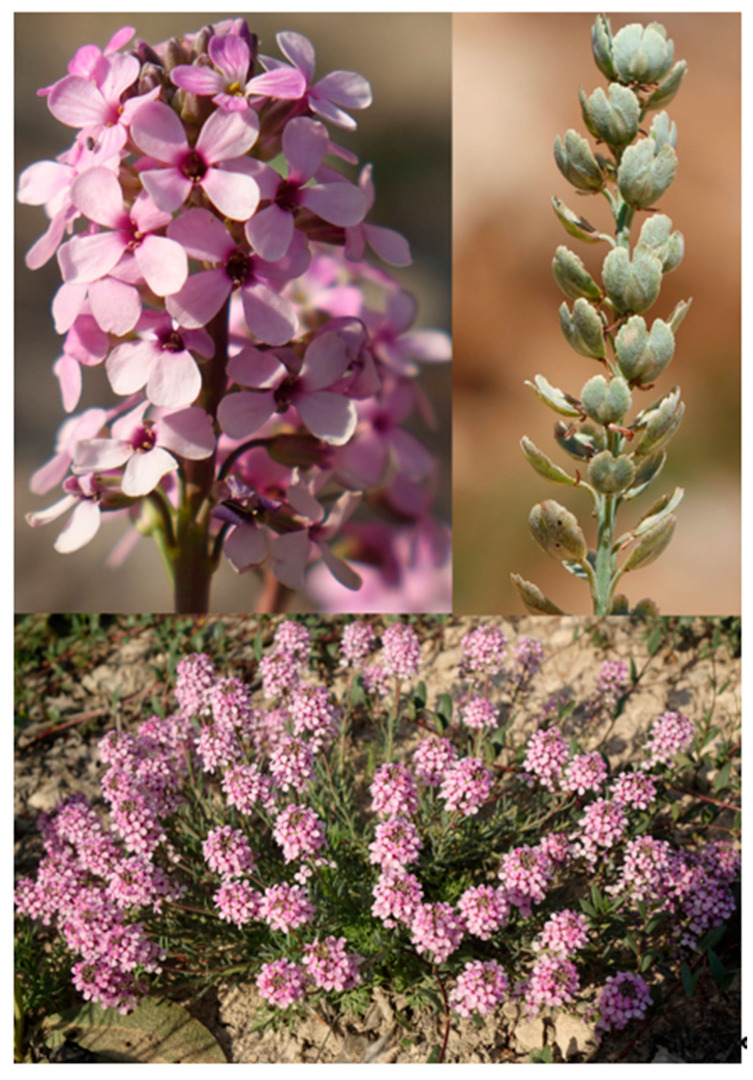
Habit, flowers and infructescence of *Aethionema beysehirense*.

#### 2.4.4. *Aethionema ermenekense* Ertuğrul & Uysal, sp. Nov. (Figure 5 and Figure 10)

*Type*: Karaman: Sarıeliler, Göktepe (Karalar) plateau, high mountain steppe, 1830 m, 19 June 2017, *K. Ertuğrul* 5367 & H. Dural (KNYA).

*Diagnosis: A. ermenekense* is similar to *A. spicatum* and *A. yildirimlii*, but it differs from *A. spicatum* by violet margined with green band sepals (vs. white margined with violet band), spatulate (vs. obovate) petals, elliptic (vs. obovate) ovary, and weakly cymbiform (vs. cymbiform) fruits with style, 0.1–0.2 mm (vs. 0.5–1 mm) long. It differs from *A. yildirimlii* by its unbranched (vs. basally branched) stems, 2.4–2.5 mm long (vs. 3–3.5 mm) sepals, elliptic (vs. ovate) ovary, 2.5–3.5 mm long (vs. 3.5–4.5 mm) wing, and style 0.1–0.2 mm long (vs. 0.3–0.5 mm) (Table 4).

Perennial, with woody caudex, multi-stemmed at the base; stems (2.5–) 4–12 cm long, erect–ascending, unbranched, glabrous, and glaucous. Leaves heteromorphic; stem leaves sub-fleshy, glaucous, subsessile or rarely sessile, alternate, oblong near to inflorescence, attenuate at base, obtuse at apex, (5–) 6–10 (–11) × 1–2.5 mm; glomerule leaves dense towards the base, fleshy, glaucous, subsessile, alternate–opposite, obovate, attenuate at base, obtuse at apex, 2.5–5 × 1.2–2.5 mm. Racemes capitate, corymbose, 0.5–1 cm long, slightly elongated in fruit. Pedicels 2–3 mm long in flowers, 3.5–4 mm in fruit, adpressed and recurved. Sepals oblong, violet margined with green band, 2.4–2.5 × 1–1.2 mm. Petals spatulate, 4.5–6 × 2.1–2.5 mm, pink, differentiated into claw and blade. Filaments free, inner not toothed, dilated at base, 1.5–1.6 mm long; outer 1–1.2 mm long; anthers yellow, apiculate, 0.5–0.8 mm long; stigma capitate, papillous; ovarium elliptic. Infructescence compact, imbricated, 1–2 cm long; fruits weakly cymbiform, dehiscent, obovate-orbicular, 7–9 × 7–10 mm, bilocular, 1-seeded per locule, cordate at base, cell 3.5–4.5 × 2.5–3 mm, septum 3.5–4.5 × 1.1–2 mm, entire or rarely undulate at margin, wing 2.5–3.5 mm long, sinus closed and 1.5–3 mm deep, style 0.1–0.2 mm long. Seed brown-gray ovate, 2.77–2.82 × 1.48–1.51, verrucate.

*Etymology*: The specific epithet was derived from the name of Ermenek district from where the first specimens of the new species were collected. The vernacular name of *A. ermenekense* was proposed as “Göktepe kayagülü” according to guidelines published by Menemen et al. [25].

*Distribution*, *habitat and ecology*: *A. ermenekense* grows on steppe, stony slopes in the Ermenek-Göktepe district of Karaman province at altitudes of 1830 m. This region is part of the Mediterranean floristic region (Figure 7).

*Additional examined specimens*: *A. ermenekense*: Türkiye. Karaman: Sarıveliler, west of Göktepe (Karalar) plateau, high mountain steppe, 1830 m, 23 April 2018, *K. Ertuğrul* 5547 (KNYA). Ermenek, Sarıveliler, Göktepe (Karalar) plateau, 1830 m, stony slopes, 15 July 2019, K. Ertuğrul 5912 (KNYA); Sarıveliler, Göktepe (Karalar) plateau, high mountain steppe, 1830 m, 21 May 2022, K. Ertuğrul 6832 (KNYA); Ermenek-Sarıvelliler, west of Göktepe (Karalar) plateau, 1830 m, 2 July 2022, H. Demirelma 3464 and E. Şirin (KNYA). *A. spicatum*: Türkiye. Adana, Pozantı, Hamidiye to Çamlıbel, clearings of *Pinus* forest, 1290 m, 7 June 2020, *K. Ertuğrul 6017 & H.Demirelma* (KNYA), Mersin, Kaynakkeşli village, around Kızılasma bridge, clearings of *Pinus* forest, 760 m, 17 May 2019, *K. Ertuğrul 5693 & H.Demirelma* (KNYA). *A. yildirimlii:* Türkiye: Konya, Derebucak, Çamlık village, around Kızıldağ, forest clearings, serpentine slopes, 1534 m., 22 May 2022, *K. Ertuğrul* 6839 (KNYA), Kızıldağ, forest clearings, serpentine slopes, 1580 m, 15 June 2021, *T. Uysal* 4358 (KNYA).

*A. ermenekense* resembles *A. spicatum* and *A. yildirimlii* in its wider stem leaves and compact infructescences. It differs from *A. spicatum* by its oblong (vs. oblanceolate–obovate) stem leaves, 1.2–2.5 mm (vs 2.5–4.5 mm) wide, glomerulate leaves 1–2.5 mm (vs. 2–4.5 mm) wide, flowering pedicels 2–3 mm (vs. 3.5–4.5 mm) long, violet margined sepals with green band (vs. white margined with violet band), spatulate petals 2.1–2.5 mm wide (vs. obovate and 1.5–2.1 mm wide), inner filaments 1.5–1.6 mm (vs. 1.8–2.5 mm) long, and elliptic (vs. obovate) ovary. It differs from *A. yildrimlii* by the unbranched stems 4–12 cm long (vs. basally branched and 12–20 cm long), stem leaves 6–11 mm (vs. 4–6 mm) long, pedicels 2–3 mm long in flower and 3.5–4 mm in fruit (vs. 3–4.5 mm in flowers and 4.5–5 mm in fruit), sepals 2.4–2.5 mm (vs. 3–3.5 mm) long, petal differentiated into claw and blade and 4.5–6 mm long (vs. undifferentiated and 6–7 mm long), inner filaments 1.5–1.6 mm (vs. 2.5–2.8 mm) long, elliptic (vs. ovate) ovary, fruit wing 2.5–3.5 mm (vs. 3.5–4.5 mm) long, and style 0.1–0.2 mm (vs. 0.3–0.5) mm long.

**Figure 10:**
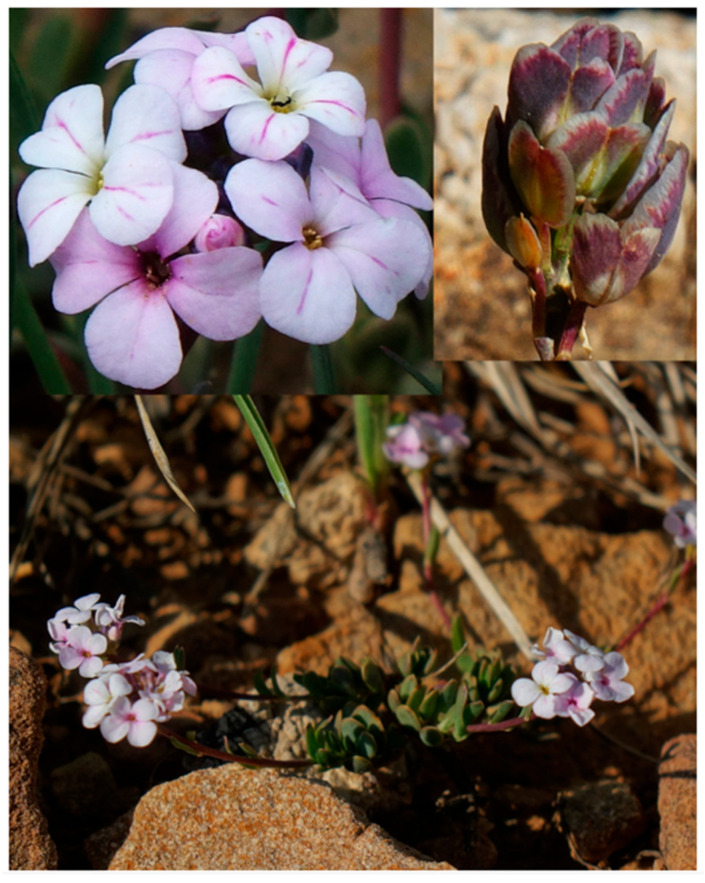
Habit, flowers and infructescence of *Aethionema ermenekense*.

## 3. Discussion and Conclusions

In Mediterranean Türkiye, different vegetations are formed under changing climatic conditions and topography [26]. Geographic, ecological, and climatic factors as well as interspecific hybridization (introgression) and polyploidy play important roles in speciation and diversification of *Aethionema* [24,27].

Mohammadin et al. [28] concluded that the center of origin of *Aethionema* is most probably along the Anatolian Diagonal, from which it spread to the Irano-Turanian region after the uplift of Iranian and Anatolian plateaus and the formation of mountain ranges in Iran and Türkiye. This concept is confirmed by the present study as well as by Ertuğrul et al. [24], revealing that two local endemics occupy an ancestral position compared to those of the Anatolian Diagonal, where half of the 55 taxa in Türkiye are distributed. It is clear, however, that the Taurus Mountains play an important role in the evolution and differentiation of the genus but not as much as the Anatolian Diagonal.

As a result of the uprise of the Zagros Mountains, different habitats appeared due to the orogeny in Iran. A similar orogenic formation occurred for Central Taurus, where numerous new habitats have contributed to the evolution of many new *Aethionema* species. The Mediterranean vegetation in Türkiye is divided into two Mediterranean biome and orobiome ecoregions, with the former extending along the Mediterranean coastline and the latter covering mountainous areas above 1000 m [26]. Ertuğrul et al. [24] indicated that ten local endemics (*A. lycium* I.A. Andersson et al., *A. demirizii* Davis & Hedge, *A. subulatum* (Boiss. & Heldr.) Boiss., *A. alanyae* H. Duman, *A. karamanicum* Ertuğrul & Beyazoğlu, *A. eunomioides* (Boiss.) Bornm., *A. compactum* (Hartvig & Strid) Yıld., *A. yıldırımlii* Kılıç, *A. thesiifolium* Boiss. & Heldr., and *A. balansae* Boiss.) are localized especially in the orobiome ecoregion (on calcareous slopes and rock crevices of rocky slopes). Serpentine rocks or their fragmented surfaces rarely provide a special habitat for some endemics, such as *A. compactum* and *A. yildirimlii*. Apart from this, Mesozoic limestone, karst landforms and reddish Mediterranean soils are spectacular habitats for some *Aethionema* taxa. For example, dolines are very special geological formations and host many relict and endemic species. They are generally found in the upper parts of the Taurus Mountains. For example, *A. ermenekense* is distributed in red soil meadows surrounded by sparsely distributed and degraded cedar forests on sloping limestone rocky areas. Several other narrow endemics (e.g., *Muscari vuralii* Bagci & Dogu, *Centaurea ermenekensis* Şirin & Uysal, *Sedum ermenekensis* Yıld. & Dinç, *Gladiolus izzet-baysalii* Eker & Sagiroglu and *Haplophyllum ermenekense* Ulukuş & Tugay) were discovered from the same region. *Aethionema uysalii* is a subalpine that grows on limestone rocky crevices east of Antalya. Recently discovered novelties such as *Bilacunaria aksekiensis* A. Duran & B. Doğan, *Polygonum uysalii* S. Makbul, Coskunç. & S. Kundakçi, *Chaerophyllum aksekiense* A. Duran & H. Duman, and *Linaria dumanii* A. Duran & Y. Menemen occupy different altitudes and habitats within two different Mediterranean ecobiomes. Thus, one can conclude that these special habitats on the Taurus Mountains accommodate many endemics, of which dozens of novelties were discovered in the last decade.

**Figure 4:**
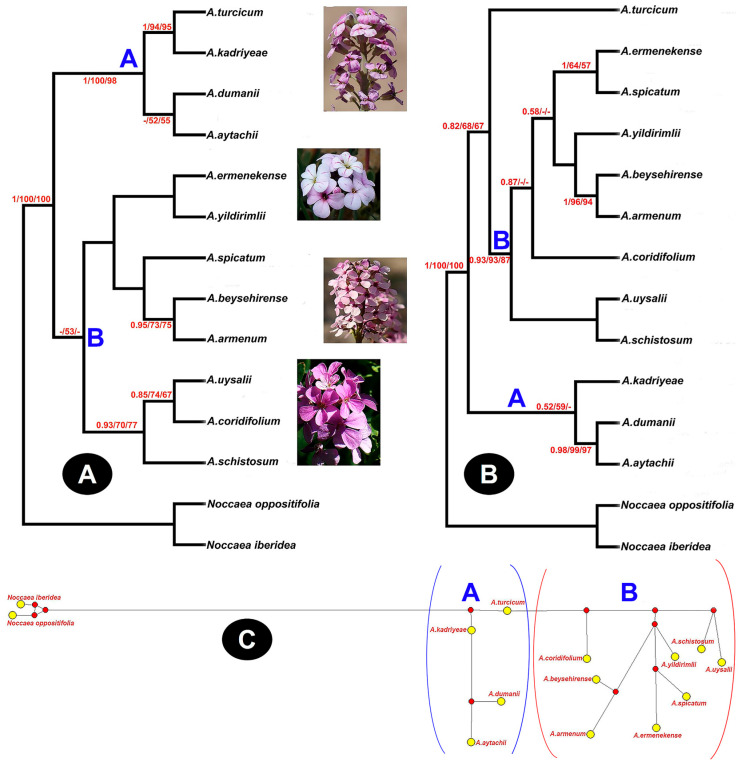
Neighbor-Joining trees showing phylogenetic relationships based on nuclear (**A**) and plastid (**B**) sequences and network analyses (**C**) based on plastid sequences. The numbers on the branch are given as posterior probability (PP) and bootstrap (BS; MP and ML), respectively (ITS; CI:0.853, RI:0.857, HI:0.147 and rpl32; CI:0.884, RI:0.889, HI:0.116). The letters A and B, indicated in blue on the phylogenetic tree, represent the major clades.

**Figure 7:**
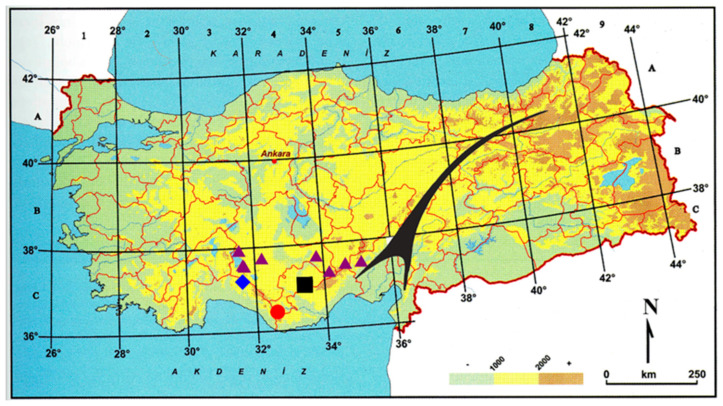
Distribution map of *A. ermenekense* (●), *A. uysalii* (♦), *A. kadriyeae* (■) and *A. beysehirense* (▲).

Interestingly, both *A. uysalii* and *A. ermenekense* are morphologically related to *A. spicatum*, but they differ in their phylogenetic relationships. *A. spicatum* is widely distributed in the Eastern Mediterranean, where it grows on limestone, serpentine rocks, or slopes in forest clearings. In ITS-based phylogenetic analyses, *A. spicatum* has been shown to exhibit a relatively weak phylogenetic affinity with *A. beysehirense* and *A. armenum*, which are distributed between the Anatolian Plateau and the Mediterranean phytogeographical region. In contrast, plastid genes (*rpl32–trnL* intron) indicate that the species displays a closer phylogenetic relationship with *A. ermenekense* compared to nuclear genomic data. Accordingly, phylogenetic relationships inferred from chloroplast DNA sequences are congruent with morphological characters. In contrast, ITS-based phylogenetic inferences are incongruent with morphology. This discordance is most plausibly explained by introgressive hybridization resulting from gene flow mediated by interspecific pollination with a sympatrically distributed species. Furthermore, network analyses provide strong evidence that *A. spicatum* and *A. ermenekense* are closely related phylogenetically, indicating a high degree of genetic affinity between the two taxa. Similarly, *A. uysalii* exhibits incongruence between maternal (chloroplast) and nuclear phylogenetic signals. Specifically, chloroplast-based inference indicates a closer evolutionary affinity to *A. schistosum* than to *A. coridifolium*, in contrast to relationships inferred from nuclear data. *A. beysehirense*, another new subalpine species, is distributed in limestone slope clearings of semi-shrub forest that display a transition from the Mediterranean orobiome to the Anatolian plateaus. Although the species is morphologically similar to *A. schistosum*, it is phylogenetically related to *A. armenum*. This species appears to be adapted to transition areas among the Mediterrenean and the Anatolian plateaus rather than the dolines. By contrast, *A. kadriyeae,* another transition zone species, is very locally distributed on limestone slopes of Kayaönü, an area with many endemics (e.g., *Linum ciliatum* Hayek, *Aethionema karamanicum*, *Polygala inexpectata* Peşmen & Erik, etc.). The phylogenetic position of all four new *Aethionema* indicates differentiation in the ITS and chloroplast sequence data, though an inconsistency occurs. While *A. kadriyeae* shares a common position with *A. dumanii* and *A. aytachii* from the gypsum areas of the Anatolian plateau in the plastid inheritance, it shows a closer relationship with *A. turcicum* according to the nuclear ITS sequences (BS and PP values above the branches of the trees). Similar incongruencies have been reported in much earlier studies, e.g., [28,29,30,31]. The ITS phylogeny is congruent with the findings of Mohammadin et al. [28] and Lenser et al. [29], supporting the delineation of the two principal clades (A and B). The *rpl32-trnL*-based phylogeny likewise corroborates the presence of these major clades, with the exception of *A. turcicum*. Furthermore, network analysis inferred from *rpl32-trnL* sequences provides additional support for the separation of these major clades (Figure 4).

Seed morphology was previously shown to have some taxonomic value in Brassicaceae, e.g., [32,33,34,35]. Bona [36] found that seed morphology in the 14 *Lepidium* L. taxa from Türkiye is tuberculate, ruminate, reticulate, reticulate-areolate, reticulate-tuberculate, or reticulate-fovate type. By contrast, Kaya et al. [37] demonstrated that the seed morphology is also useful in *Malcolmia* W.T. Aiton, *Strigosella* Boissier and *Zuvanda* Askerova (now *Plagioloba* Reichenbach) at the species level. By contrast, seed morphology was shown by Pınar et al. [38] to support the division of *Hesperis* L. into sections *Hesperis*, *Mediterreraneae*, *Pachycarpos*, *Delicate*, *Diaplictos* and *Contorta*.

In their study of 17 taxa of *Aethionema*, Pınar et al. [38] showed that seed shape, color and ornamentation are taxonomically useful in distinguishing species. Four types of seed ornamentation (reticulate, reticulate-clavate, reticulate-verrucate and verrucate) are found in *Aethionema*, and Demirpolat [39] showed that the seeds of *A. sancakense* are broadly ovoid, brown, and reticulate-verrucate. Our present results overlap significantly with the above-mentioned studies and support the value of overall seed morphology (including SEM data) in the diagnoses and recognition of the four new species. For example, *A. kadriyeae* differs from the related *A. turcicum* by having broadly ovate (vs. ovate) seeds; *A. uysalii* resembles *A. spicatum* in having ovate seeds that differ from *A. coridifolium*, which has broadly ovate seeds; and *A. beysehirense* has ovate seeds similar to *A. armenum* but differs from *A. schistosum* with broadly ovate seeds. Ovate seeds are observed in *A. ermenekense* and *A. spicatum*, while *A. yildirimlii* has broadly ovate ones. The seed color is not found useful for delimitation taxa because close species have the same seed color. All new species and their close relatives have reticulate-verrucate seed sculpture. Finally, the smallest seeds were observed in *A. turcicum*, while the largest ones are in *A. ermenekense*.

Palynological data, especially shape and sculpture, is also useful in the separation of four new taxa from their close relatives. For example, *A. kadriyeae* has prolate-spheroidal-shaped pollen grains, while the related *A. turcicum* has prolate ones. However, *A. uysalii* and close relatives *A. spicatum* and *A. coridifolium* cannot be distinguished according to pollen shape and sculpture of exine. Therefore, the aperture type can separate *A. spicatum*, but it is not useful between *A. uysalii* and *A. cordifolium*. Pollen shape is subprolate in *A. beysehirense*, as it is in *A. schistosum*, but differs from *A. armenum*, which has prolate-spheroidal. Both tetracolpate and tricolpate aperture morphology was seen in *A. beysehirense*, while *A. schistosum* had only tricolpate. Similarly, *A. ermenekense* has only tricolpate aperture that differs from its close relatives *A. yildirimlii* and *A. spicatum*, which have tricolpate-tetracolpate. Çeter et al. [40] reported that the pollen shape, size, surface ornamentation, and muri and lumina shape and size are important in distinguishing *Aethionema* species, and their results are congruent with ours.

The infructescence morphology, especially compact (vs. lose), is one of the main characters in the separation of taxa in *Aethionema*. Furthermore, fruit morphology (especially shape and base, sinus length and opening, septum and style length, cell structure and shape, wing length) is very useful in determining species boundaries, and these are discussed under each of the four new species.

## 4. Materials and Methods

Field trips were mainly conducted in 2020–2024. Approximately 1500 *Aethionema* samples were collected from different habitats throughout Türkiye. Morphological observations and measurements were carried out in the field as well as in the laboratory. Distribution maps of all the species were prepared based on records from both our fieldwork and the localities from examined herbarium specimens.

Pollen of various species was taken from herbarium materials and prepared following Wodehouse [41]. The non-acetolysis pollen grains were separated by anthers and were stained with glycerin-jelly and covered with coverslips. Pollen was observed using a Leica DM 1000 light microscope with a Canon 450 D camera (Ota City, Tokyo, Japan) and measured using Kameram 21 software (Argenit, İstanbul, Türkiye). At least 30 or more pollen were investigated per sample.

At least 20 mature fruits or seeds per sample were investigated for micromorphological analyses. For scanning electron microscope (SEM) studies, pollen and seeds were directly transferred to small aluminum stubs, and they were coated with gold aid of a sputter coater. Photographs of pollen and seeds were taken with a Zeiss Evo LS 10 microscope (Carl Zeiss NTS GmbH, Oberkochen, Germany). Refs. [38,40,41,42] were followed for palynological and seed micromorphological terminology.

For molecular phylogenetic studies, samples of various species of *Aethionema* and *Noccaea* (as outgroup) were collected from different regions of Türkiye (Table 5). Total DNA isolation was performed using the 2X CTAB method described by Doyle and Doyle [43] and modified by Soltis et al. [44] and Cullings [45]. For PCR amplification, primers were used for ITS1 and ITS4 regions [46] and *rpl32-trnL*^UAG^ [47]. Amplification conditions and procedures were carried out in accordance with previously established methodologies reported in [48] and [47], respectively. The raw sequences were edited using the Chromas Lite program. All sequences were aligned in the Bioedit program (v.7.0.5.3 version; [49]). Neighbor Joining (NJ), Parsimony and Maximum Likelihood (ML); the SYM + G and GTR + G evolutionary model was used for ITS and *rpl32-trnL*, respectively; the default option in PAUP analyses was performed in the PAUP program (v.4.0b10 version; [50]). Bootstrap analyses were performed with 1000 repetitions for the reliability of branches. Both the retention index (RI) and consistency index (CI) were given for the strict consensus trees, with the exclusion of uninformative characters. Bayesian inference (BI) of the two datasets was calculated using MrBayes v3.2.6 [51]. The best available model of molecular evolution required for Bayesian estimations of phylogeny was selected using Akaike information criteria (AIC) for both datasets as implemented in the software MrModelTest v2.2 [52]. The best fitting models for the ITS and *rpl32-trnL* datasets were TIM2ef + G and K81uf + G, respectively. Bayesian analyses for ITS and *rpl32-trnL* were conducted using random starting trees, which were then run for 6.1 × 10^4^ and 10 × 10^4^ generations, for 2 independent 4 Metropolis-coupled chain runs. For every 1000 generations that were run, only one was recorded. The run output was examined for convergence by considering the standard deviation of the split frequencies that were near 0.001. The first 1000 samples (20%) were considered to be burn-in and were removed after they were visually examined with regards to the likelihood score plots. The stationary of runs as well as the convergence between the runs were examined using Tracer software (v.1.7.0 version) [53]. Phylogenetic network analyses were performed with the Network program [54]. Bayesian (BI), Maximum Likelihood (ML), and Maximum parsimony (MP) trees based on nuclear (ITS) and plastid (rpl32-trnL) sequences are provided in the Supplementary Materials.

## Figures and Tables

**Figure 1:**
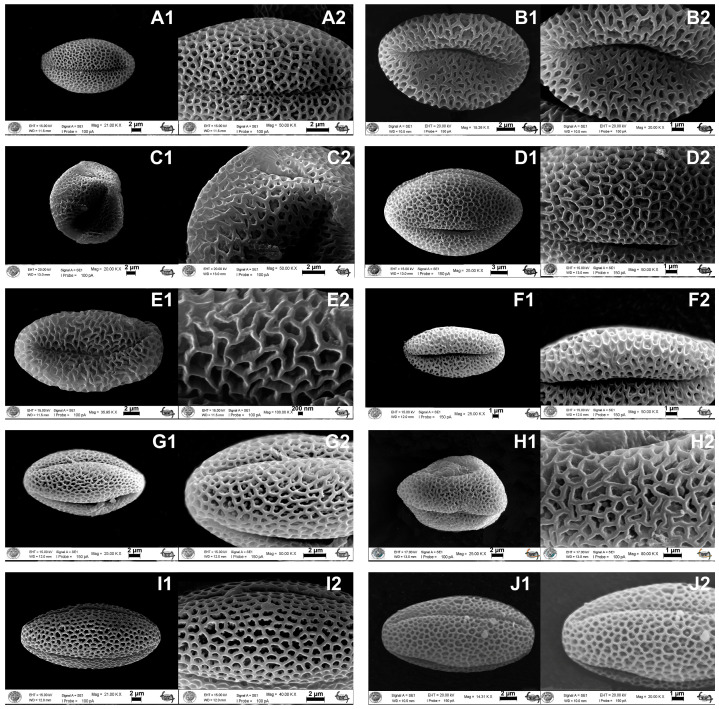
The comparative pollen morphological characters of *Aethionema* species by scanning electron microscope (SEM). (**A1**,**A2**) *A. kadriyeae.*, (**B1**,**B2**) *A. turcicum*, (**C1**,**C2**) *A. uysalii*, (**D1**,**D2**) *A. spicatum*, (**E1**,**E2**) *A. coridifolium*, (**F1**,**F2**) *A. beysehirense*, (**G1**,**G2**) *A. schistosum*, (**H1**,**H2**) *A. armenum*, (**I1**,**I2***) A. ermenekense*, (**J1**,**J2**) *A. yildirimlii*.

**Figure 2:**
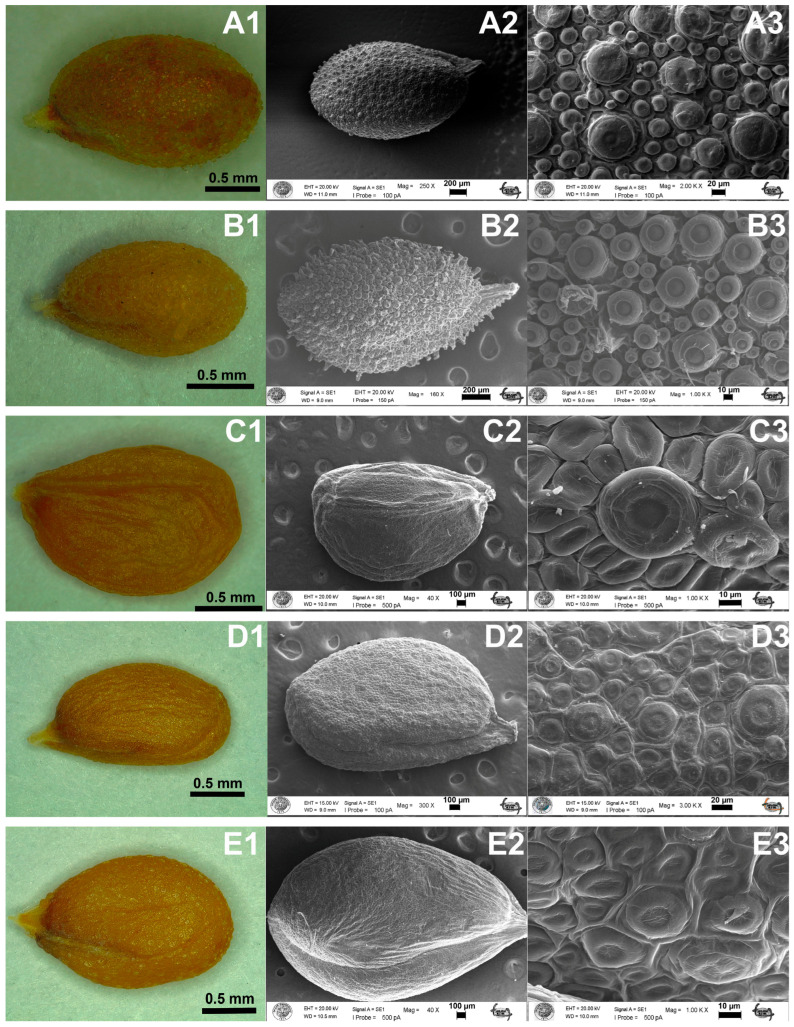
The comparative seed morphological characters of *Aethionema* species by scanning electron microscope (SEM). (**A1**–**A3**) *A. kadriyeae*, (**B1**–**B3**) *A. turcicum*, (**C1**–**C3**) *A. uysalii*, (**D1**–**D3**) *A. spicatum*, (**E1**–**E3**) *A. coridifolium*.

**Figure 3:**
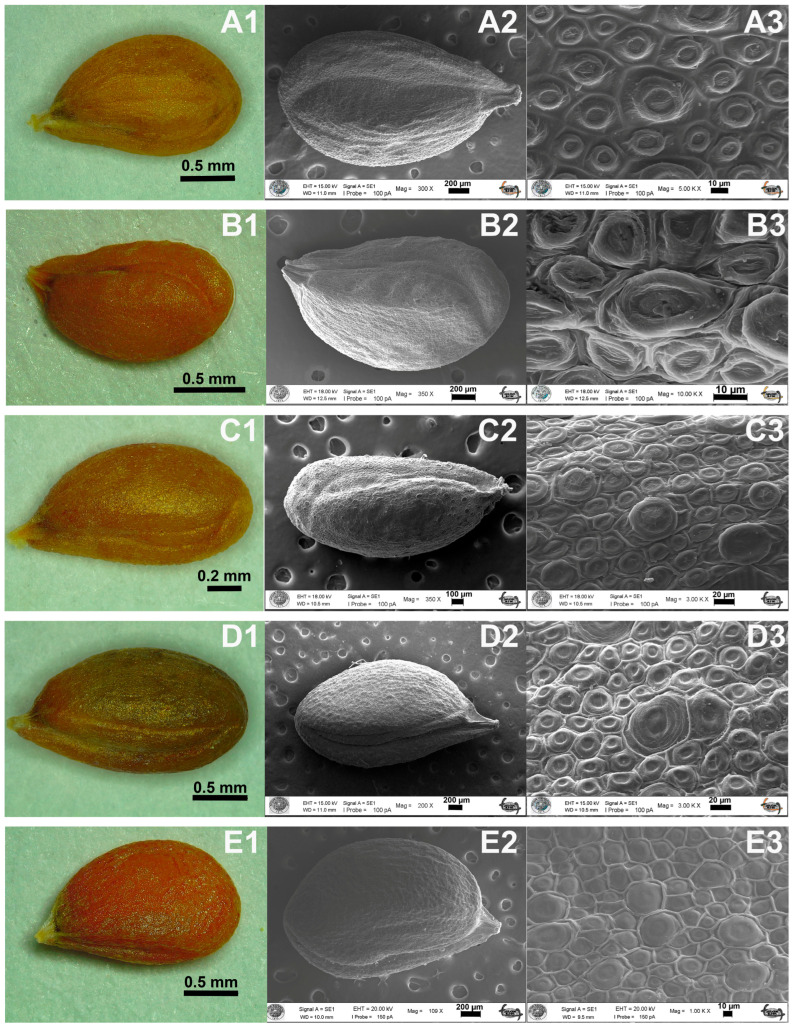
The comparative seed morphological characters of *Aethionema* species by scanning electron microscope (SEM). (**A1**–**A3**) *A. beysehirense*, (**B1**–**B3**) *A. schistosum,* (**C1**–**C3**) *A. armenum*, (**D1**–**D3**) *A. ermenekense*, (**E1**–**E3**) *A. yildirimlii*.

**Table 1:** The comparative pollen features of *Aethionema* species (values in μm, *n* = 30).

Taxa/Pollen Characters	Polar Axes	Equatorial Axes	P/E Ratio	Pollen Shape	Colpus Length (Clg)	Colpus Width (Clt)	Exine	Intine	Apocolpium	Aperture	Sculpture	Lumina
*A. kadriyeae*	20.01 ± 0.90	12.42 ± 0.42	1.15	Prolate-spheroidal	16.98 ± 1.06	3.10 ± 0.46	1.44 ± 0.07	0.62 ± 0.03	3.44 ± 0.47	Tricolpate	Microreticulate	0.87 ± 0.10
*A. turcicum*	20.04 ± 2.31	13.21 ± 1.79	1.51	Prolate	15.77 ± 1.41	3.02 ± 1.48	1.15 ± 0.04	0.59 ± 0.05	3.07 ± 1.06	Tricolpate	Reticulate	1.03 ± 0.17
*A. uysalii*	17.64 ± 1.13	15.24 ± 1.85	1.15	Prolate-spheroidal	15.21 ± 0.62	4.22 ± 0.42	1.34 ± 0.17	0.66 ± 0.06	3.18 ± 1.67	Tricolpate	Reticulate	1.19 ± 0.30
*A. spicatum*	20.44 ± 1.00	20.22 ± 0.46	1.01	Prolate-spheroidal	22.42 ± 0.48	2.12 ± 1.07	1.43 ± 0.18	0.59 ± 0.08	3.34 ± 1.15	Tricolpate-Tetracolpate	Reticulate	1.01 ± 0.26
*A. coridifolium*	16.50 ± 1.16	15.75 ± 0.82	1.04	Prolate-spheroidal	14.64 ± 0.3	5.06 ± 0.46	1.26 ± 0.20	0.62 ± 0.05	2.52 ± 0.34	Tricolpate	Reticulate	1.26 ± 0.25
*A. beysehirense*	21.14 ± 2.41	16.91 ± 3.45	1.25	Subprolate	16.09 ± 4.63	3.61 ± 0.92	1.22 ± 0.19	0.58 ± 0.05	4.34 ± 0.55	Tricolpate-Syncolpate	Microeticulate	1.59 ± 0.25
*A. schistosum*	16.41 ± 1.04	13.66 ± 1.03	1.20	Subprolate	14.49 ± 0.93	3.12 ± 0.19	0.94 ± 0.21	0.48 ± 0.04	4.19 ± 0.11	Tricolpate	Microeticulate	1.19 ± 0.29
*A. armenum*	18.12 ± 0.78	16.23 ± 0.50	1.11	Prolate-spheroidal	14.66 ± 0.71	5.71 ± 1.10	1.15 ± 0.22	0.54 ± 0.06	2.20 ± 0.59	Tricolpate-Syncolpate-Tetracolpate	Microreticulate	0.83 ± 0.15
*A. ermenekense*	17.64 ± 1.13	15.24 ± 1.85	1.15	Prolate-spheroidal	15.21 ± 0.62	4.22 ± 0.42	1.34 ± 0.17	0.66 ± 0.06	3.18 ± 1.67	Tricolpate	Reticulate	1.19 ± 0.30
*A. yildirimlii*	15.98 ± 0.99	15.56 ± 0.87	1.02	Prolate-spheroidal	14.52 ± 1.37	5.67 ± 0.38	1.36 ± 0.17	0.57 ± 0.11	4.71 ± 0.74	Tricolpate-Tetracolpate	Microreticulate	0.76 ± 0.09

**Table 2:** The comparative seed characteristics of *Aethionema* species (*n* = 20).

Taxa/Seed Characters	Length (mm)	Width (mm)	L/W Ratio	Seed Shape	Seed Color
	Mean	Mean			
*A. kadriyeae*	2.17	1.26	1.76	Broadly ovate	Light-brown and brown
*A. turcicum*	1.65	0.86	1.84	Ovate	Light-brown and brown
*A. uysalii*	2.11	1.42	1.86	Ovate	Light-brown
*A. spicatum*	2.29	1.25	1.75	Ovate	Light-brown and brown
*A. coridifolium*	2.87	1.73	1.66	Broadly ovate	Light-brown
*A. beysehirense*	2.33	1.25	2.03	Ovate	Light-brown
*A. schistosum*	2.02	1.18	1.70	Broadly ovate	Brown
*A. armenum*	2.11	1.00	2.03	Ovate	Light-brown
*A. ermenekense*	2.79	1.49	1.86	Ovate	Brown-grey
*A. yildirimlii*	2.68	1.47	1.75	Broadly ovate	Brown

**Table 3:** Comparison of morphological characters of *A. kadriyeae*, *A. uysalii* and related taxa.

Characters	*A. kadriyeae*	*A. turcicum*	*A. uysalii*	*A. spicatum*	*A. coridifolium*
Stem leaves	elliptic, attenuate at base	ovate, rounded at base	oblanceolate, attenuate at base	obovate, attenuate at base	linear–oblanceolate, attenuate at base
Raceme	cylindrical	cylindrical	capitate	capitate	cylindrical
Infructescence	lax	lax	compact	compact	lax
Sepals (mm)	oblong, 2.5–3.5 × 1.5–1.6	oblong, 2.7–3.1 × 1.3–1.6	oblong, 3–3.5 × 0.8–1.2	oblong, 2–4 × 1–1.5 (–2)	oblong, 2.7–3.5 × 1.1–1.8
Petals (mm)	obovate, 6.5–7.5 × 2.5–4	obovate, 5–6.5 × 2.5–4	spatulate–obovate, 7.5–8 × 2.7–5	obovate, 4–6 × 1.5–2.1 (–3)	obovate, 5.5–6 × 3.2–4
Inner filaments (mm)	not toothed, 3–3.5	toothed, 2–3	not toothed, 1.8–2.8	not toothed, 1.8–2.5	not toothed, 2.5–3
Pedicel in fruit (mm)	erect, 5–5.5	horizontal–recurved, 4–7	adpressed, 4.5–5	ascending, 3.5–6	erect–recurved, 3.5–6.5
Ovary	elliptic	oblong	elliptic	obovate	ovate-orbicular
Fruits (mm)	oblong–obovate, cordate at base, 6–8 × 5.5–7	oblong, sagittate at base, 8.5–11 × 8–9	obovate, cordate at base, 7.5–9.5 × 8–11	obovate–orbicular, cordate at base, 6–11 × 5–11	ovate–orbicular, cordate at base, 6–8.5 × 5.5–6.5
Wing (mm)	1.5–2.5	2.5–3	4.5–5.5	2–3.5	2–3.5
Cell (mm)	3.5–5.5 × 2–2.5 (–3.5)	5.5–7 × 2–3	3–4 × 2.5–3.5	3–3.5 × 2–3.5	2.5–4 × 2–3.5
Septum (mm)	4–5.5 × 1–2	5–7 × 1.5–2.1	3.5–4 × 1–1.3	3–5 × 0.9–1	3.5–4.5 × 1.4–2.5
Sinus (mm)	open, 0.5–0.8	closed, 0.7–1.1	open, (3.5–) 4–5	closed, 2–4	closed, 1–2.5
Style (mm)	1–1.7	1–1.5	0.5	0.5–1	0.2–0.3

**Table 4:** Comparison of morphological characters of *A. beysehirense*, *A. ermenekense* and related taxa.

Characters	*A. beysehirense*	*A. schistosum*	*A. armenum*	*A. ermenekense*	*A. yildirimlii*
Stem leaves	linear, rarely involute and falcate, attenuate at base	linear, attenuate at base	linear-oblanceolate, attenuate at base	oblong, attenuate at base	oblanceolate, attenuate at base
Raceme	cylindrical	capitate	capitate	capitate	capitate
Infructescence	lax	compact	lax	compact	compact
Sepals (mm)	oblong rarely obovate, 2.5–3.5 × 1–1.5	oblong–obovate, 2.5–3 × 0.8–1.2	oblong, 2–3.5 × 1–1.7	oblong, 2.4–2.5 × 1–1.2	oblong, 3–3.5 × 1–1.5
Petals (mm)	spatulate, 5–7 × 2–4	spatulate, 4.5–7 × 2.5–4	oblanceolate or obovate, 4–6 × 1.8–3	spatulate, 4.5–6 × 2.1–2.5	spatulate-obovate, 6–7 × 2.4–3
Inner filaments (mm)	not toothed, 1.7–2.7	not toothed, 1.8–2.6	not toothed, 2.2–3.5	not toothed, 1.5–1.6	not toothed, 2.5–2.8
Pedicel in fruit (mm)	adpressed–recurved, 4–6	adpressed–recurved, 4.5–5	adpressed–ascending, 3–5	adpressed and recurved, 3.5–4	adpressed—recurved, 4.5–5
Ovary	obovate	obovate–oblong	oblong	elliptic	ovate
Fruits (mm)	obovate, cordate at base, 6.5–7.5 × 5–7.5	orbicular, cordate at base, 7–10.5 × 9–12	obovate rarely oblong, cordate at base, 4–6.5 × 3–5	obovate-orbicular, cordate at base, 7–9 × 7–10	obovate-orbicular, cordate at base, 7–8.5 × 7–9.5
Wing (mm)	2–4	4–5	1–2	2.5–3.5	3.5–4.5
Cell (mm)	3–4.5 × 2–3	3.5–4.2 × 2–3.5	2–4 ×1–2.2	3.5–4.5 × 2.5–3	3–3.5 × 2.2–3
Septum (mm)	3.5–5 × 1–1.6	3–4 × 0.8–1.2	3–4 × 0.7–1.5	3.5–4.5 × 1.1–2	3.5–4.5 × 0.9–1.1
Sinus (mm)	closed, 0.8–1	closed, 3–3.5	open, 0.5–1	closed, 1.5–3	closed, 2–3
Style (mm)	0.5–1	0.2–0.5	0.5–1	0.1–0.2	0.3–0.5

**Table 5:** Voucher specimens for the ITS and *rpl32*-*trnL* study.

Taxa	Collection No	ITS Genebank	*rpl32-trnL* Genebank
*A. turcicum*	*K.Ertuğrul* 5754	MW791189 (Uysal et al. In: Ertuğrul et al. [22])	PV166694 (In this study)
*A. kadriyeae*	*K.Ertuğrul* 6339	PV156495 (In this study)	PV166695 (In this study)
*A. dumanii*	*K.Ertuğrul* 5755	MW791190 (Uysal et al. In: Ertuğrul et al. [22])	PV166696 (In this study)
*A. aytachii*	*K.Ertuğrul* 5757	MW791188 (Uysal et al. In: Ertuğrul et al. [22])	PV166697 (In this study)
*A. ermenekense*	*K.Ertuğrul* 5912	PV156496 (In this study)	PV166698 (In this study)
*A. yildirimlii*	*H.Demirelma* 2917(ITS), *K.Ertuğrul* 5849 (*rpl32*)	PV156497 (In this study)	PV166699 (In this study)
*A. spicatum*	*K.Ertuğrul* 6016	PV156498 (In this study)	PV166700 (In this study)
*A. uysalii*	*K.Ertuğrul* 6124(ITS), *K.Ertuğrul* 6123 (*rpl32*)	PV156499 (In this study)	PV166701 (In this study)
*A. coridifolium*	*K.Ertuğrul* 6557	PV156500 (In this study)	PV166702 (In this study)
*A. beysehirense*	*K.Ertuğrul* 6905	PV156501 (In this study)	PV166703 (In this study)
*A. armenum*	*K.Ertuğrul* 6521	PV156502 (In this study)	PV166704 (In this study)
*A. schistosum*	*K.Ertuğrul* 6851	PV156503 (In this study)	PV166705 (In this study)
*Noccaea oppositifolia*	*K.Ertuğrul* 6836	PV156504 (In this study)	PV166706 (In this study)
*N. iberidea*	*K.Ertuğrul* 6818	PV156505 (In this study)	PV166707 (In this study)

## Data Availability

The original contributions presented in this study are included in the article. Further inquiries can be directed to the corresponding authors.

## Supplementary Materials

The following supporting information can be downloaded at: https://www.mdpi.com/article/10.3390/plants15081180/s1. S1: Bayesian (BI), Maximum Likelihood (ML), and Maximum parsimony (MP) trees showing phylogenetic relationships based on nuclear (ITS) and plastid (rpl32-trnL) sequences.

## References

[1] Çetintaş O., Sözen M. The Importance of Taurus Mountains in Terms of Turkey Zoogeography and Biodiversity. Proceedings of the 3rd International Congress on Zoology and Technology.

[2] Davis P.H., Davis P.H., Harper P.C., Hedge I.C. (1971). Distribution patterns in Anatolia with particular reference to endemism. Plant Life of South-West Asia.

[3] Karl R., Koch M.A. (2013). A world-wide perspective on crucifer speciation and evolution: Phylogenetics, biogeography and trait evolution in tribe Arabideae. Ann. Bot..

[4] Mutun S., Dinç S. (2019). The Anatolian Diagonal and paleoclimatic changes shaped the phylogeography of *Cynips quercus* (Hymenoptera, Cynipidae). Ann. Zool. Fenn..

[5] Manafzadeh S., Staedler Y.M., Conti E. (2016). Visions of the past and dreams of the future in the Orient: The Irano-Turanian region from classical botany to evolutionary studies. Biol. Rev..

[6] Djamali M., Baumel A., Brewer S., Jackson S.T., Kadereit J.W., López-Vinyallonga S., Mehregan I., Shabaniang E., Simakova A. (2012). Ecological implications of *Cousinia* Cass. (Asteraceae) persistence through the last two glacial-interglacial cycles in the continental Middle East for the Irano-Turanian flora. Rev. Palaeobot. Palynol..

[7] Atalay I., Altunbaş S., Khan A.A., Coşkun M. The Mountain Ecology of the Taurus Mountains and Its Effects on Nomadism. Proceedings of the International Geography Symposium on the 30th Anniversary of TUCAUM.

[8] Atalay I. (2006). The effects of mountainous areas on biodiversity: A case study from the northern Anatolian Mountains and the Taurus Mountains. Grazer Schriften Geogr. Und Raumforsch..

[9] Çiplak B. (2003). Distribution of Tettigoniinae (Orthoptera, Tettigoniidae) bush-crickets in Turkey: The importance of the Anatolian Taurus Mountains in biodiversity and implications for conservation. Biodivers. Conserv..

[10] German D.A., Hendriks K.P., Koch M.A., Lens F., Lysak M.A., Bailey C.D., Mummenhoff K., Al-Shehbaz I.A. (2023). An updated classification of the Brassicaceae (Cruciferae). PhytoKeys.

[11] POWO (2026). Plants of the World Online. Facilitated by the Royal Botanic Gardens, Kew. https://powo.science.kew.org/.

[12] Hedge I.C., Hedge I.C., Rechinger K.H. (1968). *Aethionema* R. Br. & *Moriera* Boiss. Flora Iranica.

[13] Al-Shehbaz I.A. (2012). A generic and tribal synopsis of the Brassicaeae (Cruciferae). Taxon.

[14] Moazzeni H., Al-Shehbaz I.A., German D.A., Assadi M., Mueller J., Joharchi M.R., Memariani F. (2018). A taxonomic revision of the genus *Aethionema* s.l. (Brassicaceae) in Iran. Phytotaxa.

[15] Hedge I.C., Davis P.H. *Aethionema* R. Br. Flora of Turkey and the East Aegean Islands.

[16] Ertuğrul K., Güner A., Aslan S., Ekim T., Vural M., Babaç M.T. (2012). *Aethionema* W.T. Aiton. Turkey’s List of Plants (Vascular Plants).

[17] Karabacak O., Öztürk M., Duran A. (2013). *Aethionema anatolica* (Brassicaceae), a new species from south Anatolia, Turkey. Ann. Bot. Fenn..

[18] Yıldırımlı Ş., Kılıç Ö. (2016). New infrageneric taxa and species of *Aethionema* W.T. Aiton (Brassicaceae) and their current key from Turkey. Herb J. Syst. Bot..

[19] Yıldırımlı Ş., Kılıç Ö. (2018). A new species of *Aethionema* (Brassicaceae), *A. adiyamanense* from Turkey. Herb J. Syst. Bot..

[20] Yıldırımlı Ş., Kılıç Ö. (2019). A New *Aethionema* (Brassicaceae) A. sancakense p. p. and a new description of *A. adiyamanense* from Turkey. Herb J. Syst. Bot..

[21] Kandemir A., Aytaç Z., Fişne A.Y. (2017). *Aethionema erzincanum* (Brassicaceae), a new species from Turkey. Ann. Bot. Fenn..

[22] Ertuğrul K., Hamzaoğlu E., Demirelma H., Uysal T., Bozkurt M., Şirin E., Çitak B.Y., Al-Shehbaz I.A. (2021). *Aethionema aytachii* (Brassicaceae): A new species from central Anatolia, Turkey. Turk. J. Bot..

[23] Öztürk D. (2022). *Aethionema gypsicola*, a new crucifer species from inner Anatolia, Turkey. Phytotaxa.

[24] Ertuğrul K., Uysal T., Demirelma H., Şirin E., Bozkurt M., Yilmaz Çitak B. (2026). Novelties in the genus *Aethionema* (Brassicaceae) in Türkiye: Resurrections, new combination and status, and new synonyms. Turk. J. Bot..

[25] Menemen Y., Aytaç Z., Kandemir A. (2016). Türkçe Bilimsel Bitki Adlandırma Yönergesi. Bağbahçe Bilim Derg..

[26] Atalay I., Efe R., Soykan A., Cravins E., Atalay Ö. (2008). Mediterranean Ecosystems of Turkey: Ecology of The Taurus Mountains. Part I, Chapter One. Environment and Culture in the Mediterranean Region.

[27] Bozkurt M., Ertuğrul K., Uysal T., Şekeroğlu N., Süntar İ., Bahadırlı N.P. Karyomorphological Studies of three Endemic *Aethionema* (Brassicaceae) Species in Turkiye. (Full paper). Proceedings of the 12th International Mediterranean Symposium on Medicinal and Aromatic Plants (MESMAP–12), Proceedings Book.

[28] Mohammadin S., Peterse K., van de Kerke S.J., Chatrou L.W., Dönmez A.A., Mummenhoff K., Pires J.C., Edger P.P., Al-Shehbaz I.A., Schranz M.E. (2017). Anatolian origins and diversification of *Aethionema*, the sister lineage of the core Brassicaceae. Am. J. Bot..

[29] Lenser T., Graeber K., Çevik Ö.S., Adigüzel N., Dönmez A.A., Kettermann M., Mayland-Quellhorst S., Mérai Z., Mohammadin S., Nguyen T.-P. (2016). Developmental control and plasticity of fruit and seed dimorphism in the Brassicaceae *Aethionema*. Plant Physiol..

[30] Koch M., Dobeš C., Kiefer C., Schmickl R., Klimeš L., Lysak M. (2007). Supernetwork identifies multiple events of plastid trnF(GAA) pseudogene evolution in the Brassicaceae. Mol. Biol. Evol..

[31] Franzke A., German D.A., Al-Shehbaz I.A., Mummenhoff K. (2009). Arabidopsis’s family ties: Molecular phylogeny and age estimates in the Brassicaceae. Taxon.

[32] Zohary M. (1948). Carpological studies in Cruciferae. Palest. J. Bot..

[33] Vaughan J.G., Whitehouse J.M. (1971). Seed structure and the taxonomy of the Cruciferae. Bot. J. Linn. Soc..

[34] Appel O., Al-Shehbaz I.A., Kubitzki K., ve Bayer C. (2002). Cruciferae. The Families and Genera of Vascular Plants.

[35] El Naggar S.M. (2005). Seed coat micro-sculpturing and the systematic of the Egyptian Brassicaceae (Magnoliopsida). Flora Mediter..

[36] Bona M. (2013). Seed coat microsculpturing of Turkish *Lepidium* (Brassicaceae) and its systematic application. Turk. J. Bot..

[37] Kaya A., Ünal M., Özgökçe F., Doğan B., Martin E. (2011). Fruit and seed morphology of six species previously placed in *Malcolmia* (Brassicaceae) in Turkey and their taxonomic value. Turk. J. Bot..

[38] Pınar N.M., Adıgüzel N., Geven F. (2007). Seed Coat Macrosculpturing In Some Turkish *Aethionema* R. Br. (Brassicaceae). Pak. J. Bot..

[39] Demirpolat A. (2022). Anatomical, Palynological and Seed Surface Characteristics of *Aethionema sancakense* Yıld. & Kılıc (Brassicaceae). Avrupa Bilim Ve Teknol. Derg. Özel Sayı.

[40] Çeter T., Geven F., Şahin A.A., Çeter S. (2018). Examination of pollen morphology of some *Aethionema* (Brassicaceae), from Turkey. Commun. Fac. Sci. Univ. Ank. Series C..

[41] Wodehouse R.P. (1935). Pollen Grains.

[42] Punt W., Hoen P., Blackmore S., Nilsson S., Le Thomas A. (2007). Glossary of pollen and spore terminology. Rev. Palaeobot. Palynol..

[43] Doyle J.J., Doyle J.L. (1987). A rapid DNA isolation procedure for small quantities of fresh leaf tissue. Phytochem. Bull. Bot. Soc. Am..

[44] Soltis D.E., Soltis P.S., Collier T.G., Edgerton M.L. (1991). Chloroplast DNA Variation Within and Among Genera of The Heuchera Group (Saxifragaceae): Evıdence For Chloroplast Transfer And Paraphyly. Am. J. Bot..

[45] Cullings K.W. (1992). Design and testing of a plant-specific PCR primer for ecological and evolutionary studies. Mol. Ecol..

[46] White T.J., Bruns T., Lee S., Taylor J., Innis M.A., Gelfand D.H., Sninsky J.J., White T.J. (1990). Amplification and direct sequencing of fungal ribosomal RNA genes for phylogenetics. PCR Protocols: A Guide to Methods and Applications.

[47] Shaw J., Lickey E.B., Schilling E.E., Small R.L. (2007). Comparison of whole chloroplast genome sequences to choose noncoding regions for phylogenetic studies in angiosperms: The Tortoise and The Hare III. Am. J. Bot..

[48] Garcia-Jacas N., Uysal T., Romashchenko K., Suárez-Santiago V.N., Ertuğrul K., Susanna A. (2006). *Centaurea* revisited: A molecular survey of the Jacea group. Ann. Bot..

[49] Hall T.A. (1999). BioEdit: A user-friendly biological sequence alignment editor and analysis program for Windows 95/98/NT. Nucleic Acids Symp. Ser..

[50] Swofford D.L. (2002). PAUP*. Phylogenetic Analysis Using Parsimony (* And Other Methods), ver. 4.0b10.

[51] Ronquist F., Teslenko M., Mark P.V.D., Ayres D.L., Darling A., Ohna S.H., Larget B., Liu L., Suchard M.A., Huelsenbeck J.P. (2012). MrBayes 3.2: Efficient Bayesian Phylogenetic Inference and Model Choice Across a Large Model Space. Syst. Biol..

[52] Nylander J.A.A. (2004). Computer Program Distributed by the Author. MrModel Test.

[53] Rambaut A., Drummond A.J., Xie D., Baele G., Suchard M.A. (2018). Posterior summarisation in Bayesian phylogenetics using Tracer 1.7. Syst. Biol..

[54] Bandelt H.J., Forster P., Sykes B.C., Richards M.B. (1995). Mitochondrial portraits of human populations. Genetics.

